# Unexpected High Digestion Rate of Cooked Starch by the Ct-Maltase-Glucoamylase Small Intestine Mucosal α-Glucosidase Subunit

**DOI:** 10.1371/journal.pone.0035473

**Published:** 2012-05-01

**Authors:** Amy Hui-Mei Lin, Buford L. Nichols, Roberto Quezada-Calvillo, Stephen E. Avery, Lyann Sim, David R. Rose, Hassan Y. Naim, Bruce R. Hamaker

**Affiliations:** 1 Whistler Center for Carbohydrate Research, Department of Food Science, Purdue University, West Lafayette, Indiana, United States of America; 2 United States Department of Agriculture (USDA), Agricultural Research Service, Children’s Nutrition Research Center, Department of Pediatrics, Baylor College of Medicine, Houston, Texas, United States of America; 3 Ontario Cancer Institute and Department of Medical Biophysics, University of Toronto, Toronto, Ontario, Canada; 4 Department of Biology, University of Waterloo, Waterloo, Ontario, Canada; 5 Institute of Physiological Chemistry, University of Veterinary Medicine Hannover, Hannover, Germany; University of Canterbury, New Zealand

## Abstract

For starch digestion to glucose, two luminal α-amylases and four gut mucosal α-glucosidase subunits are employed. The aim of this research was to investigate, for the first time, direct digestion capability of individual mucosal α-glucosidases on cooked (gelatinized) starch. Gelatinized normal maize starch was digested with N- and C-terminal subunits of recombinant mammalian maltase-glucoamylase (MGAM) and sucrase-isomaltase (SI) of varying amounts and digestion periods. Without the aid of α-amylase, Ct-MGAM demonstrated an unexpected rapid and high digestion degree near 80%, while other subunits showed 20 to 30% digestion. These findings suggest that Ct-MGAM assists α-amylase in digesting starch molecules and potentially may compensate for developmental or pathological amylase deficiencies.

## Introduction

Starch is the major dietary carbohydrate for humans. It consists of two glucans, amylose and amylopectin. Amylose is composed of long linear chains of D-glucose units linked by α-1,4-glycosidic linkages with few branches; while amylopectin has higher molecular weight with shorter linear glucans linked by α-1,4-linkages and is highly branched by α-1,6-linkages [1]. To generate dietary glucose from starchy foods, salivary and pancreatic α-amylase and four intestinal mucosal α-glucosidase activities, C- and N-terminal maltase-glucoamylase (MGAM) and sucrase-isomaltase (SI), are employed. α-Amylase (enzyme class EC 3.2.1.1.) hydrolyzes starch endowise at inner α-1,4 linkages and produces linear maltooligosaccharides with α-configuration [2]. It does not hydrolyze α-1,6 linkages, and some neighboring α-1,4 linkages, and all the branch linkages remain as branched oligosaccharides. α-Amylases from human saliva and pancreas have similar hydrolysis patterns. Both α-amylases produce maltose (G2) preferentially from reducing residues of maltotetraose (G4), maltopentaose (G5) and maltohexaose (G6) and essentially do not act on maltotriose (G3) [2]. After a prolonged incubation with a large amount of porcine pancreatic α-amylase, there is produces negligible glucose from reducing residues of G3 [2], [3].

The four mucosal α-glucosidase activities are associated with the two membrane-bound MGAM (EC 3.2.1.20 and 3.2.1.3) and SI (EC 3.2.148 and 3.2.10) complexes. Each protein complex contains two catalytic subunits: an N-terminal subunit that is anchored to the enterocyte membrane and a C-terminal luminal subunit [4]. Here we compare for the first time the four individual subunit activities for direct digestion of cooked starch. All four catalytic subunits are classified under the glycosyl hydrolysate Family 31 (GH31) [5], [6] and have certain similarities in their amino acid sequence. Both N-terminal MGAM and SI and the respective C-terminal subunits are more closely related in sequence to one another than to their corresponding subunits within the same complex [7], because MGAM and SI activities were evolved by duplication of an ancestral gene [6]. Each subunit of the MGAM and SI complexes has maltase [8] and maltotriase activities and hydrolyzes α-1,4 glycosidic linkages from non-reducing ends [4], [9]. SI, which is 40 to 50 times more abundant in amount than MGAM [10], is responsible for 80% maltase and maltotriase activities in the human body [11] though MGAM digests short linear oligomers more rapidly than SI [6], [12]. Developmentally, the four mucosal activities are expressed only after weaning in rodents but are present from birth in humans. There is a developmental delay in pancreatic amylase activity secretion in both rodents and humans [13], [14], [15], [16], [17]. This developmental delay has been used as the reason for delaying feeding of cereals in the first months of life [16]. Physiologically, MGAM is more highly active and, for instance, may be important to meet the oxidative needs of children’s brain metabolism, whereas the slower but more abundant SI may moderate glucose delivery in high glycemic starchy diets [10]. In addition to α-1,4 linkage activity, MGAM only has a marginal role in hydrolysis of α-1,6 linkages in the human body [9]. SI is responsible for almost all isomaltase activity [6] with the N-terminal subunit known as the “isomaltase subunit” [18]. On the other end, the C-terminal SI subunit is responsible for sucrose hydrolysis and is known as the “sucrase subunit” [9].

A confusing issue regarding starch digestion is lack of consideration of the role of the mucosal α-glucosidases in regulating digestion rate. Conventional *in vitro* digestibility methods are based on the susceptibility of starch to porcine pancreatic α-amylase [19], [20], [21], [22], [23], [24], [25], [26], [27], [28], [29]. Variations of this method include use of thermo-stable α-amylase to substitute for pancreatic α-amylase [22], [24], [29], [30], [31], addition of salivary α-amylase [27], [28], pullulanase [20], protease [22], [24], [30], [31], and/or invertase [21], and pre-treatment of substrates with lichinase and α-glucosidase [22]. Starch is nutritionally classified into rapidly digestible starch, slowly digestible starch and resistant starch by the hydrolysis of the combination of pancreatic α-amylase and fungal glucoamylase [20], [21], [32], [33]. Little attention has been given to the contribution of the gut mucosal α-glucosidases in starch digestibility. The prevailing viewpoint seems to be that α-amylase is the determinant of digestion rate of starch and that gut mucosal α-glucosidases do not digest big molecules and rapidly convert α-amylase products into glucose.

Here we ask the question whether gut mucosal α-glucosidases participate and contribute to digestion of gelatinized starch molecules. Our research group has investigated gut mucosal α-glucosidase digestion at various starch structural levels. Recombinant human Nt-MGAM was found to be capable, albeit at a very low rate, to digest intact starch granules to glucose [34]. Mucosal α-glucosidase digestion was also examined at the α-limit dextrin (LDx) level. α-LDx is the starch product that cannot be further digested by α-amylase. Three mucosal enzyme subunits digested the highly branched structure of α-LDx, and the four subunits showed individual digestion patterns [35]. Mgam null mice showed a reduction in α-LDx digestion by one-half, suggesting that Mgam is important to starch digestion [36], [37]. α-Amylase certainly amplified glucogenesis in an *in vitro* system [34]. *In vivo*, α-amylase amplified both wild type and MGAM null mice mucosal glucogenesis [37]. Thus, both *in vitro* and *in vivo* systems indicate a considerable contribution of mucosal α-glucosidases in starch digestion at various structural levels. Here our objective was to investigate whether gut mucosal α-glucosidases are capable to digest gelatinized starch without the aid of α-amylase. In this study, gelatinized normal maize was incubated with individual recombinant mucosal α-glucosidase subunits of varying amounts and digestion periods for the purpose of exploring their potentially larger role in starch digestion than previously thought.

## Materials and Methods

### Recombinant Mucosal α*-*glucosidases

Recombinant human Nt-MGAM, Ct-MGAM and Nt-SI and recombinant mouse Ct-Mgam and Ct-Si were employed in this research. The production of recombinant human Nt-MGAM and Nt-SI was performed as described previously [12], [37]. The methods of producing recombinant human Ct-MGAM and mouse Ct-Mgam are described in Appendix S1. All human (H-1320); animal (AN-1577) experiments; and molecular analyses/recombinant expressions (D-952) were approved by the respective committees of Baylor College of Medicine.

### Starch Digestion with Individual Mucosal α-glucosidases

Normal maize (Tate & Lyle, Decatur, IL ) was dispersed in a 10 mmol/L phosphate buffer (pH 7.0, 10 mg of starch dry mass/mL), and then cooked in a boiling water bath with stirring at about 200 rpm for 30 min. Phosphate buffer was chosen to maintain the constant pH environment. Gelatinized starch was cooled down to 37°C before adding individual mucosal α-glucosidases. An aliquot of cooked starch (10 µL) was transferred to microcentrifuge tubes and incubated with individual mucosal α-glucosidases (5, 10, 20, 30 and 100 units) for different time intervals (1, 2, 3, 4, 5, 6, 12, and 24 h) at a water bath set at 37°C and 80 rpm. Glucosidases were inactivated by heating in a boiling water bath for 10 min. The released glucose amount was determined by the glucose oxidase-peroxidase (GOPOD) assay [38] (Megazyme International Ireland Ltd., Wicklow, Ireland). The digestion experiments were done in triplicate.

To test the digestion capability on large starch molecules, recombinant α-glucosidases activities were normalized based on amounts required to hydrolyze 50 mmol/L maltose in 10 mmol/L phosphate buffer. Assays were done in triplicate. One unit of activity was defined as the amount of glucose (µg) that is released from 10 µL of 50 mmol/L maltose at 37°C in 5 min. The released glucose amount was determined by the GOPOD assay. Statistical analysis was performed using one-way ANOVA and Tukey’s multiple comparision test. Significance was considered at P <0.05. To compare the starch digestive capability of each enzyme subunits, the applied enzymes (30 units) were further converted to specific activity, the released glucose amount (mg) per pmol protein in the enzyme preparation.

### Stability of Mammal Mucosal α-glucosidases Activities

Mucosal α-glucosidases were incubated at 37°C water bath, and aliquot (1 or 2 µL) was taken at different time intervals to react with 10 µL malotse (50 mmol/L) for activity assay described above.

## Results

### Starch Digestion with Individual Mucosal α-glucosidase

**Figure 1:**
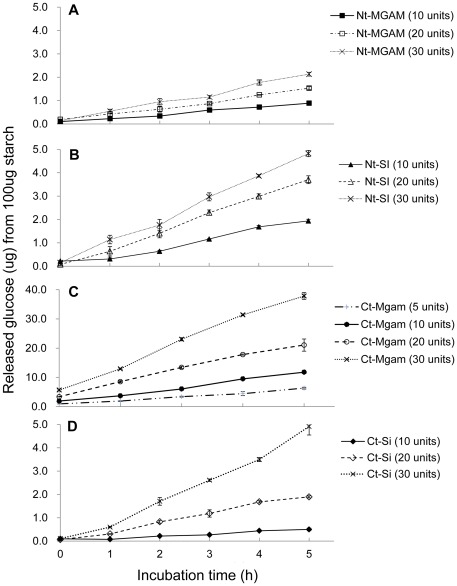
Direct digestion profiles of mucosal α-glucosidases with different activity units and incubation times. Cooked normal maize (100 µg) was incubated with mucosal glucosidase including human Nt-MGAM, mouse Ct-Mgam, human Nt-SI, and mouse Ct-Si at 37°C for 5 h. Three to four enzyme amounts, 5, 10, 20 and 30 units were applied in the system. The released glucose amount was determined by the GOPOD method. Values are means ± SD in triplicate analysis.

**Figure 2:**
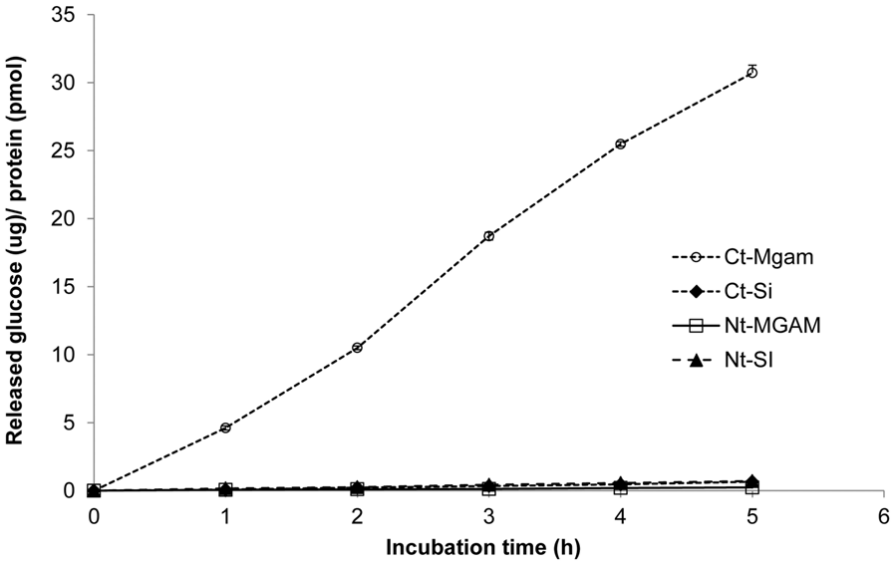
Direct starch digestive capability of individual mucosal α-glucosidases. Enzyme amount, 30 units, in Figure 1 was converted to specific activity, amount (µg) of released glucose per pmol protein.

The production of glucose from cooked normal maize starch digested with the four individual mucosal α-glucosidases, human Nt-MGAM, human Nt-SI, mouse Ct-Mgam, and mouse Ct-Si, is shown in Fig. 1. Normal maize starch, a common food ingredient, contains approximately 25% amylose. Without α-amylase participation all four individual mucosal α-glucosidase subunits digested cooked starch and released some amount of glucose and at different rates. All four subunits increased the digestion extent when more enzyme units were applied and/or were incubated for a longer time. At 30 units α-glucosidase incubated 5 h, human Nt-MGAM reached 2.1%, human Nt-SI reached 4.8%, mouse Ct-Mgam reached 37.8%, and mouse Ct-Si reached 4.9% digestion. Digestion degree was calculated as:
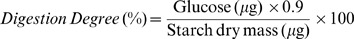



Mouse Ct-Mgam was comparably much more active in directly digesting cooked starch, even at low enzyme units (5 units), than other subunits. In Figure 2, starch digestive capability of each α-glucosidase subunit is compared based on the specific activity, and Ct-Mgam again showed the highest starch digestive capability.

**Figure 3:**
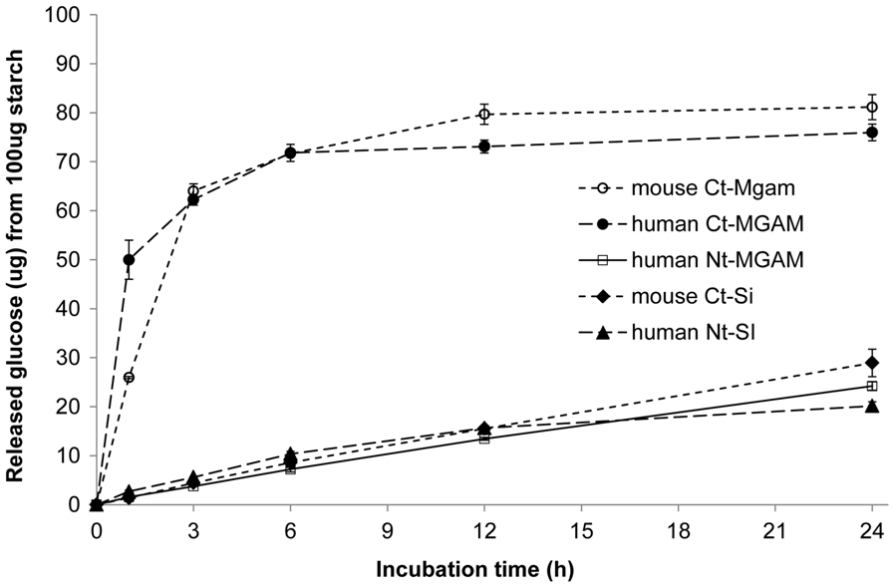
The direct digestion profiles of mucosal α-glucosidase of 100 activity units. Cooked normal maize (100 µg) was incubated with mucosal α-glucosidase including human Nt-MGAM, human Ct-MGAM, mouse Ct-Mgam, human Nt-SI, and mouse Ct-Si at 37°C for 24 h. Relatively high enzyme amount, 100 units, was applied in the system. The release glucose amount was determined by GOPOD method. Values are means ± SD in triplicate analysis.

To test the upper limitation of mucosal α-glucosidase digestion of cooked starch, 100 enzyme units were added of each subunit and digested for a prolonged period. In this test, recombinant human Ct-MGAM was included. All four subunits increased digestion extent, and both mouse and human Ct-MGAM reached a plateau level after 6 h with a small increase to 81 and 76% digestion after 24 h, respectively (Figure 3). The other three subunits increased in digestion extent with increasing incubation time reaching 20–29% digestion after 24 h. Mouse and human Ct-MGAM showed similar cooked normal maize starch digestion extent and rate.

### Stability of Individual Mucosal α-glucosidases Activities

**Figure 4:**
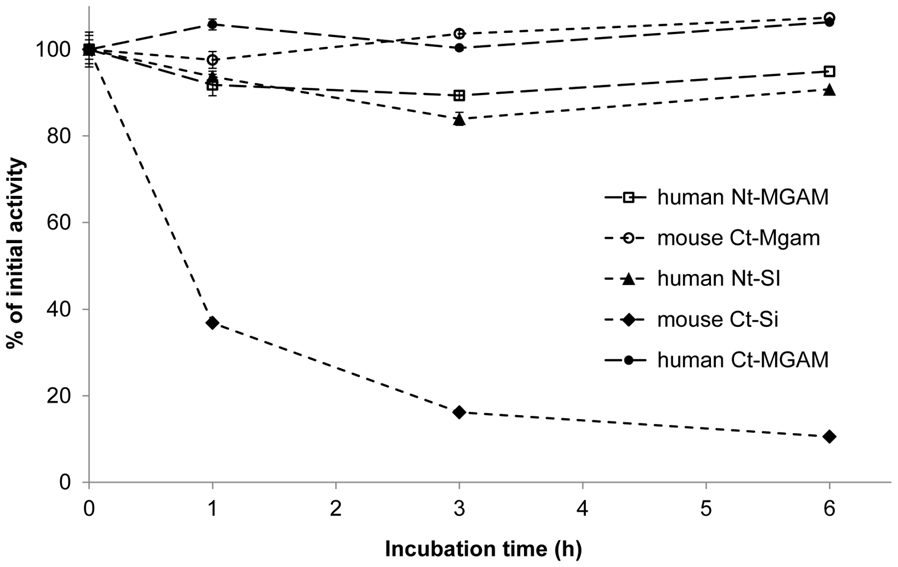
The stability of mucosal α-glucosidases activity. Mucosal α-glucosidases including human Nt-MGAM, human Ct-MGAM, mouse Ct-Mgam, human Nt-SI, and mouse Ct-Si at 37°C. Aliquot was taken at 1, 3 and 6 h, and maltose (50 mmol/L) was applied as the substrate to test the activity. The remaining activity is the percent of the initial activity; the activity is defined as the amount (µg) of released glucose per pmol protein. Values are means ± SD in triplicate analysis.

The glucosidase activity was determined by using maltose as a substrate, and the stability of maltase activity during the digestion progress was determined (in Fig. 4). Mouse Ct-Si activity dropped about 37% activity during the first hour, and with only about 10% activity remaining after six hours; human Nt-SI activity decreased about 10% activity during the incubation period, indicating some instability of SI over long incubation time in the *in vitro* system used in this study. The other two subunits maintained stable activity in this system over a 6 h period.

## Discussion

This research reveals, notably, that the each subunit of the gut mucosal α-glucosidases directly digests gelatinized starch to some degree without α-amylase pre-hydrolysis. Unexpected was the considerable hydrolysis of gelatinized starch molecules by mucosal Ct-MGAM with about 50% *in vitro* digestion in the first hour and later reaching nearly 80%. Why does Ct-MGAM have such high digestion capability on gelatinized starch molecules? One explanation may be the broad activity shown in pig MGAM studies. The MGAM complex isolated from intestinal mucosa was shown to have wide ranging activity on various α-glycosidic linkages including α-1,2 of kojibiose, α-1,3 of nigerose, α-1,4 of maltooligosaccharides, α-1,5 of leucrose, and α-1,6 of isomaltose [39], [40]. However, pig MGAM preferentially cleaves α-1,4 glycosidic linkages and only showed negligible cleavage of α-1,6 linkages [39]. It also hydrolyzes linear sugar alcohols (e.g., maltitol) to a small degree, but not branched sugar alcohols such as α-D-glucopyranosyl-1,6-mannitol [39]. Our previous work showed only Ct-MGAM digests branched substrates of α-LDx, opposed to Nt-MGAM which only digests short linear maltooligosaccharides [35]. Our findings here suggest that recombinant Ct-MGAM, from both human and mouse sources, have broad activity and high digestion capability on gelatinized starch molecules.

In further understanding Ct-MGAM’s high starch degrading activity, a review of the current knowledge of its hydrolysis mechanism is useful. Ct-MGAM is a well known exo-hydrolytic enzyme while α-amylase has endo-hydrolytic activity with a role to quickly break down starch molecules. In light of such high activity on gelatinized starch, it is reasonable to speculate that Ct-MGAM has endo-activity contributing to the high digestion, but this is not supported by digestion mechanism studies. Based on the subsite theory [41], the MGAM complex binds substrates via consecutive subsites, each of which interacts with a single glucose residue by hydrogen bonding and van der Waals interactions. The MGAM complex has four subsites, and the cleavage occurring between subsites 1 and 2 [42]. The number starts at the subsite that binds the glucose residue from the non-reducing end. Subsite 1 has very low affinity and makes it impossible to have endo-hydrolytic properties such as involved in transglycosylation, condensation or multiple attacks [42]. Although MGAM does not have endo-hydrolytic activity, other studies suggest that MGAM can bind large substrates, thus supporting our finding that Ct-MGAM fairly effectively digests gelatinized starch molecules. The MGAM complex has two catalytic sites [42], and it was known over a decade ago that one subunit can bind both maltose and larger maltooligosaccharides. Thus, it was proposed that MGAM has two substrate-enzyme binding modes, maltose- and maltooligosaccharide-binding modes [39], [42], [43]. Furthermore, when the enzyme binds maltooligosaccharides, the enzyme conformation may change from a maltose-binding mode to a maltooligosaccharide-binding mode [42], [43]. Our use of individual recombinant subunits confirms that from its high digestion capability, Ct-MGAM is the subunit that binds both maltose and large molecules. Another piece of evidence that Ct-MGAM binds large molecules comes from substrate inhibition studies. The presence of high concentration of G3 and G4 inhibited MGAM complex activity and was related to an enzyme conformational change [42]. The inhibition, so called “brake effect” in mucosal digestion, was later found to occur only at Ct-MGAM [12]. Kinetic studies also showed that Ct-MGAM is the subunit responsible for the high activity of immunoprecipitated human MGAM complex on various α-glucans [10], [12]. Studies of amino acid sequence alignment found Ct-MGAM has an extra 21 amino acid residues compared to the Nt-subunits. The extra residues positioned near the opening of catalytic site makes Ct-MGAM likely to form more glucose binding subsites to digest larger substrates [7]. Collectively, the broad activity and capability to bind large α-glucan substrates may account for the high degrading activity of Ct-MGAM on gelatinized starch molecules.

The other three recombinant subunits to a lower degree digested gelatinized starch molecules without α-amylase pre-hydrolysis. For Nt-MGAM, this is related to its ability to digest only linear oligomers [35] and at a relatively lower rate than Ct-MGAM (or the immunoprecipitated MGAM complex) [12]. The SI complex, as is true for the MGAM complex, has two catalytic subunits (centers) [44], but only has two rather than four glucosyl binding subsite types for α-1,4 glucans [45]. This may be the reason for the lower activity of SI. The “break effect” noted above is related to the conformational change from binding maltooligosaccharides and was only found at Ct-MGAM, but not the two subunits of SI [12]. This further supports the view that SI lacks ability to bind large molecules and results in the observed low digestion of the gelatinized starch molecules. The documented direct digestion of cooked starch by Ct-MAM suggests that the recommendation that cereals be delayed until α-amylase activity matures may need to be re-examined.

Regarding debranching activity, Nt-SI is the subunit responsible for isomaltase activity and, as well, hydrolyzes maltose [45]. Isomaltose does not compete with maltose for binding to the enzyme [45] and, thus, may have different binding modes for linear and branched structures. Nt-SI not only hydrolyzes isomaltose and panose, it hydrolyzes the linear α-1,6-isomaltooligosaccharides as well [18]. However, this subunit did not hydrolyze glycogen [46], [47], which shows its difference from other amylo-1,6-glucosidases, such as fungal glucoamylase. In our *in vitro* system, Nt-SI hydrolyzed gelatinized starch around 20% without aid of other enzymes. Thus, apparently Nt-SI hydrolytic activities at α-1,4 and α-1,6 linkages digest starch to some degree.

The current study brings forth the issue of the different as well as similar roles of α-amylase and mucosal α-glucosidases in starch digestion, and in particular how α-amylase and Ct-MGAM act on gelatinized starch molecules. The initial stage of starch hydrolysis occurs in the oral cavity, and the final stages of digestion is at the small intestine membrane, and one must reconsider the long held view of the sequential relationship of α-amylase first digesting starch molecules and mucosal α-glucosidases reducing only those products to glucose. It is interesting to note that α-amylase has been reported to participate in starch digestion when in contact with the luminal surface [47], [48], [49]. In light of our data, perhaps a better view of starch digestion is that, on the brush border membrane surface, both α-amylase and Ct-MGAM efficiently hydrolyze large molecules. Thus, both α-amylase and Ct-MGAM provide favored substrates for the mucosal α-glucosidase subunits, including Ct-MGAM itself. The implications of this work are that Ct-MGAM needs to be considered as a significant starch-degrading enzyme in normal starch digestion and in pancreatic deficiency states. Additionally, we speculate that Ct-MGAM could be a candidate mucosal subunit for targeted inhibition to moderate dietary glucose generation.

## Supporting Information

Appendix S1 **The appendix presents the method of producing recombinant human Ct-MGAM and mouse Ct-Mgam.**
(DOCX)
