# Supplementary material for: Unexpected High Digestion Rate of Cooked Starch by the Ct-Maltase-Glucoamylase Small Intestine Mucosal α-Glucosidase Subunit
Source: PLoS One. 2012 May 1;7(5):e35473. doi: 10.1371/journal.pone.0035473 (PMC3341394; doi:10.1371/journal.pone.0035473)
Supplement: Appendix S1 — The appendix presents the method of producing recombinant human Ct-MGAM and mouse Ct-Mgam. (DOCX) [file pone.0035473.s001.docx]

# **APPENDIX S1**

**Recombinant mucosal α*-*glucosidases.**

Our human MGAM nucleotide sequence has been listed as GenBank accession number AF016833. Gene-specific primers were designed from sequenced clones. Because of the 6,513 nucleotide length of the full cDNA, a truncated MGA-P1a, N-terminal domain 2,584-bp fragment of cDNA, was ligated into expression vectors and induced in *E. coli*. Expressed induced and peptides were confirmed by immunoblotting with HMA mAbs. The amino acids (87–954) of expressed construct MGAM-P1A2, lack the N-terminal cytosolic domain, the transmembrane domain, the O-glycosylated stalk region [5]. MGA-P1a was next cloned into a Drosophila pMT-BiPV5- His vector and expressed as an enzymatically active 868 residue peptide [7].The human coding sequences have been reported to GenBank for MGAM (AF016833.2). The GeneAmp XL kit (Applied Biosystems, Foster City, CA) was used for long PCR. Using the MGAM Origene_SC303516 as template, the PCR forward primer was *TACGTAATAGCATAAAGATAAGGGATGAAGAA* and reverse was *TACGTACCCAGAGTGCTTATCCACGTCAA* (Child Health Research Center Core Labortary, Baylor Colledge Medicine, Houston, TX, BCM/CHRC Core Lab). A SnaB I restriction site was inserted at the 5’ end of each primer. Amplicons of the expected size were separated on 1.5% TAE agarose gel, bands isolated, purified using QIAquick gel extraction kit (Qiagen, Chatsworth, CA), and ligated into pT7-blue T-vector, and transformed into NovaBlue *E. coli* (Novagen, Madison, WI). Clones were screened by gene specific PCR and gel electrophoresis and bands harvested using Plasmid Mini-Prep Kit (Qiagen). After digesting with SnaBI the clones were selected by predicted size and again harvested as above. DNA was sequenced to select error free inserts (Applied Biosystems, 373A automated sequencer).

The confirmed insert was ligated into pAcGP67 baculovirus vector (BD Biosciences, San Diego, CA) and named pAcGP67 His-Ct-MGAM-8. The pAcGP67 vector was modified to code ten histamines (His-1) at the 3’ end of expressed proteins. The quality of resulting constructs was confirmed by SnaBI, HaeII double digest, gel purification, harvesting and sequencing as above. These inserts were ligated into dephosphorylated pAcGP67B_His-1 plasmid and used to transform NovaBlue Singles competent cells. Colonies were screened with gene specific PCR primers, BamHI, Hind III double digestion, gel isolation, harvest and orientation confirmed by sequencing as above. These pAcGP67 His-Ct-MGAM-8-6 constructs were used to infect ten cultures of Sf9 and then High Five™ cell line cells (Invitrogen) grown in the Insectagro FIVE™ Serum-Free/Protein-Free Medium (Invitrogen).

The secreted proteins from these multiple cultures were captured using anti HisTag monoclonal antibody (Novagen) coated 96 well plates from EMD Biosciences (Gibbstown, NJ) and screened for maltase enzyme activity using the trisglucose oxidase real-time assay described previously [12]. The proteins from the most active culture were then produced as a 4 L batch and purified on His-SelectÒ Nickel Affinity columns (Sigma-Aldridge) by elution with imidazole. The purified recombinant proteins had the expected MGAM specific bands on Western blots and the enzyme activities are reported here.

**Recombinant mouse mucosal α*-*glucosidases: Ct-Mgam & Ct-Si.** The mouse coding sequences have been reported to GenBank for Mgam (EU073529) and for Si (EU937530). Using wild-type Mouse jejunal RNA template RT with SuperScript III/RNaseOUT mix (Invitrogen, Carlsbad, CA) was done. The RT Primer for Si was Oligo-dT -Adaptor-Primer and for Mgam the reverse primer below. The Mgam PCR forward primer was 2432-*ACAACAGCCTGCCACAACCAC* and reverse was 6215-*TCCAACTCCTTCCATCAGCTCTCTC* and the Si forward was 2645-*ATCTCACATTCAACCTCGGCAAA* and reverse 5373-*CCAGCTGATTTGTATTGGTTCATCA* (BCM/CHRC Core Lab). A SnaB I restriction site was inserted at the 5’ end of each SI primer and Mgam possessed unique restriction sites (EcoICR I; Taq I) in the sequence. Long PCR was done as described above. Amplicons bands were isolated and transformed into *E. coli .* Clones were screened by PCR and bands harvested. After digesting with restriction enzymes clones were selected by predicted size and again harvested. DNA in the selected bands was sequenced to select error free inserts. The confirmed inserts were named pAcGP67 His**–**CtSI and pAcGP67 His-CtMgam. The quality of resulting constructs was confirmed by Snab I in SI or EcoICR I in Mgam combined with Bgl I . These pAcGP67 His**–**CtSi and pAcGP67 His-CtMgam constructs were separately co-transfected into Sf9 insect cells, and ten isolates from each were analyzed for their ability to produce enzyme activity. Si-C14 and Mgam-N2 viral isolates that were used to infect insect cells were selected and batch produced recombinant enzymes purified as above.
